# Comment on “Surgery for Intraductal Papillary Mucinous Neoplasms of the Pancreas: Preoperative Factors Tipping the Scale of Decision-Making”

**DOI:** 10.1245/s10434-022-11774-z

**Published:** 2022-04-12

**Authors:** Zhenlu Li, Mao Li, Weiming Hu, Huimin Lu

**Affiliations:** Department of Pancreatic Surgery, West China Hospital, Sichuan University, No. 37, Guoxue Alley, Chengdu, 610041 Sichuan Province China

Dear Editor,

We read with interest the article “Surgery for Intraductal Papillary Mucinous Neoplasms (IPMN) of the Pancreas: Preoperative Factors Tipping the Scale of Decision-Making” by Marchegiani et al.^1^ We would like to congratulate the authors for their innovative work to develop a preoperative, disease-specific tool for patients undergoing surgery for IPMN. However, we need to express some of our opinions.

First, Table 3 shows postoperative outcomes with two groups distributed by type of surgery (pancreaticoduodenectomy (PD), distal pancreatectomy (DP), and total pancreatectomy (TP)) and by final pathology (low-grade dysplasia (LGD) and high-grade dysplasia/invasive (HGD/inv) cancer) but provided only one *P* value, which was confusing. We speculated about the *P* values in Table 3 for comparison between LGD and HGD/inv, whereas there were no *P* values for comparison among the three different surgical treatment groups. Were intergroup comparisons for postoperative outcomes among the three types of surgery (TP versus DP, TP versus PD, and PD versus DP) necessary for further decision tree analysis.^2^

Second, the authors integrated PD and TP into one group and concluded the same risk factors for severe surgical complications and a classification tree model for PD/TP, which might not be appropriate. Because the major morbidity after TP was lower than that after PD (19.8% versus 23.5%), clinically relevant postoperative pancreatic fistula (0% versus 15.2%) and postoperative mortality (0% versus 4.5%) were notably lower. In addition, consideration of recurrence and long-term survival after surgery for IPMN, TP, and PD might have different operation indications.^2,3^ Therefore, we think the authors should respectively provide predictors of severe surgical complications and a classification tree for PD and TP.

Overall, we thank the authors for their valuable work on developing a preoperative, disease-specific tool to predict surgical morbidity for IPMNs. In addition, to make personalized clinical decisions for surgical candidates and surgical types, preoperative risk evaluation of postoperative recurrence and long-term survival for IPMN are needed in the future.
